# Clinical Results of Mentor MemoryGel Xtra Breast Implants From the GLOW Clinical Trial

**DOI:** 10.1093/asj/sjad272

**Published:** 2023-08-23

**Authors:** Amy Alderman, David Caplin, Dennis C Hammond, Alexandra Keane, Jay Turetzky, William J Kane

## Abstract

**Background:**

Mentor MemoryGel Xtra breast implants (Mentor Worldwide LLC, Irvine, CA) were designed to maintain the soft, natural feel of MemoryGel implants while increasing fullness and projection and minimizing wrinkling, rippling, and related complications through optimization of shell gel-fill.

**Objectives:**

To measure 3-year safety and effectiveness of MemoryGel Xtra breast implants in the Mentor MemoryGel and MemoryShape Combined Cohort Clinical Study.

**Methods:**

Participants were implanted with MemoryGel Xtra breast implants in a prospective, multicenter clinical trial. Rates of complications and reoperations were analyzed to assess device safety and BREAST-Q was employed to assess device effectiveness.

**Results:**

Two hundred eighty-seven females receiving MemoryGel Xtra breast implants were enrolled. Complication rates in the primary augmentation cohort included rates of 1.5% for implant-related reoperation, 2.3% for explantation, and 1.5% for Baker grade III or IV capsular contracture. For the revisional augmentation cohort, these rates were 2.8% for implant-related reoperation, 4.3% for explantation, and 3.0% for capsular contracture. For the primary reconstruction cohort, these rates were 12.0% for implant-related reoperation, 12.3% for explantation, and 7.3% for capsular contracture. For the revisional reconstruction cohort, these rates were 7.1% for capsular contracture, with zero implant-related reoperations or explantations. There were no reports of infection or implant malposition or displacement in any of these cohorts. Each cohort showed significantly improved satisfaction with breasts and psychosocial and sexual well-being at 1 year following the primary procedure.

**Conclusions:**

These data are consistent with legacy clinical data for MemoryGel and provide the first published safety and effectiveness data regarding the use of MemoryGel Xtra breast implants for breast augmentation and reconstruction.

**Level of Evidence: 4:**

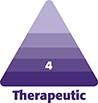

Mentor MemoryGel Xtra Silicone Gel-Filled Breast Implants (Mentor Worldwide LLC, Irvine, CA) were designed to optimize implant performance through optimal silicone gel fill volume. This enables greater device projection than with previous MemoryGel devices while maintaining the natural feel of MemoryGel.^1^ This precision filling is intended to produce the added benefits of reducing the incidence of device wrinkling and rippling and the influence of these complications on capsular contracture and shell failure. We have previously discussed the benchtop testing methodologies utilized to create MemoryGel Xtra.^1^ Here we present the 3-year clinical data for the Mentor MemoryGel Xtra cohort of the Mentor MemoryGel and MemoryShape Combined Cohort Clinical Study. This is the first prospectively collected clinical data on Mentor MemoryGel Xtra breast implants.

## METHODS

### Study Design

The MemoryGel and MemoryShape Combined Cohort Clinical Study (NCT02919592) is a prospective, open label study in which postapproval clinical performance and safety of MemoryShape and MemoryGel breast implants produced by Mentor Worldwide LLC were evaluated, as indicated for primary or revisional breast augmentation and primary or revisional breast reconstruction. The study includes both smooth and Siltex microtextured devices. IRB approval for each site was obtained from Sterling IRB, University of Tennessee Health Science Center IRB, or Providence Health and Services IRB. Implant patients were enrolled in 4 cohorts (*n* = 3171): primary breast augmentation (*n* = 2396), revisional breast augmentation (*n* = 488), primary breast reconstruction (*n* = 226), and revisional breast reconstruction (*n* = 61). In order to meet enrollment criteria, a subset of females receiving MemoryShape breast implants were retrospectively enrolled and will be followed prospectively within the study to the 10-year anniversary of their index breast implant procedure. Participants who met requirements for other aesthetic procedures, excluding silicone implantation of any kind, were enrolled as comparators. The primary objective was the 10-year estimated cumulative incidence of local complications. Secondary objectives included rates of occurrence of connective tissue disease, cancer, suicide, lactation complications, and suspected or confirmed rupture; as well as rheumatological and neurological signs and symptoms.

Inclusion criteria for prospectively enrolled patients in the study included females receiving MemoryShape or MemoryGel breast implants for augmentation purposes who were at least 22 years old or a candidate for reconstruction, who signed an acknowledgment of informed consent and authorization to disclose health information and release medical records, had a valid email address and access to the Internet to complete online questionnaires, and agreed to answer baseline questions, authorize return of explanted device(s) to Mentor, and comply with study follow-up requirements.

Exclusion criteria included active infection anywhere in the body, a confirmed diagnosis of connective tissue disease or neurological disease at the time of study entry, pregnancy or nursing at the time of study entry, planning on or undergoing bariatric surgery, or a plan to undergo experimental procedures or procedures with investigational products or devices during the study period that could compromise the results of this study. An additional exclusion criterion for augmentation patients was a history of cancer of any kind other than non-melanoma skin cancer. For reconstruction patients this criterion was a history of cancer of any kind other than non-melanoma skin cancer or adequately treated breast cancer. For the MemoryGel Xtra–specific analyses included herein, all study participants with at least 1 MemoryGel Xtra device were included. All participants had had these devices implanted at least 3 years previous to the data cutoff date.

Baseline and operative data were collected at the beginning of the study, and follow-up data will be collected annually through 10 years using online patient questionnaires. All study patients will be seen by the investigator surgeon at any standard-of-care office visits, and relevant adverse events will be captured in the study database.

Data presented were entered into the study database from February 12, 2016, through December 9, 2022. Because this is an ongoing study, results are subject to change.

### Effectiveness Analyses

Effectiveness was measured in this study with the BREAST-Q (2006 preoperative and postoperative Augmentation and Reconstruction Modules) in a subset of the 1-, 5-, and 10-year follow-up questionnaires. This report focuses on the preoperative and 1-year postoperative BREAST-Q data. The Wilcoxon signed rank test was performed to test for statistical significance between timepoints.

### Safety Analyses

All patients undergoing implantation with a study device were included in the safety population. If a study device was explanted, data up to and including the date of the explantation were included in all analyses. Per the study protocol, patients with explanted devices, whether replaced or not, were expected to remain in the study and continue to complete their annual questionnaires, which were included in the analyses. Incidence of postoperative complications was analyzed with the Kaplan-Meier method. All event rates presented here were at the participant level (as opposed to the implant level), unless otherwise specified.

### Photography

Patient photographs were provided by the authors. All photographs presented were done so with the permission of the involved surgeons and patients. All implants in these representative patients were inserted in the submuscular plane.

## RESULTS

### Demographics, Operative Characteristics, and Follow-up

A total of 287 females received at least 1 MemoryGel Xtra breast implant in the study, including 151 primary augmentation, 76 revisional augmentation, 44 primary reconstruction, and 16 revisional reconstruction patients. Both smooth and textured Siltex devices were included, although approximately 95% of the devices were smooth. All of these participants underwent implant surgery at least 3 years ago. Follow-up at the 3-year time point was 81.1% across all cohorts with the MemoryGel Xtra implants. To present a snapshot of complications that surgeons and patients can expect with MemoryGel Xtra at 3 years after implantation, data collected beyond 3-year follow-up has been excluded, other than reports of death, BIA-ALCL (breast implant–associated anaplastic large cell lymphoma), or SCC (squamous cell carcinoma). The median age (range) for each cohort was as follows: primary augmentation 34.0 (20.4-66.8), revisional augmentation 43.3 (26.0-76.5), primary reconstruction 48.0 (23.9-72.7), and revisional reconstruction 53.6 (31.6-67.7). Demographic characteristics for the study participants who received MemoryGel Xtra implants are provided in Table 1. Operative characteristics and 3-year follow-up rates for each cohort are provided in Table 2.

**Table 1.** Demographic Characteristics^a^

Characteristic	Augmentation		Reconstruction	
	Primary	Revisional	Primary	Revisional
	(*n* = 151)	(*n* = 76)	(*n* = 44)	(*n* = 16)
Median age, years	34.0	43.3	48.0	53.6
Age range, years	20.4-66.8	26.0-76.5	23.9-72.7	31.6-67.7
Median BMI, kg/m^2^	22.5	21.9	24.4	23.3
Race				
Asian, Non-Hispanic	6 (4.0%)	4 (5.3%)	0 (0%)	0 (0%)
Black or African American, Non-Hispanic	5 (3.3%)	1 (1.3%)	3 (6.8%)	1 (6.3%)
Native Hawaiian or Other Pacific Islander, Non-Hispanic	0 (0%)	1 (1.3%)	0 (0%)	1 (6.3%)
Other, Hispanic	12 (8.0%)	6 (7.9%)	2 (4.6%)	1 (6.3%)
Other, Non-Hispanic	5 (3.3%)	3 (4.0%)	0 (0%)	0 (0%)
White/Caucasian, Hispanic	9 (6.0%)	2 (2.6%)	2 (4.6%)	1 (6.3%)
White/Caucasian, Non-Hispanic	114 (75.5%)	59 (77.6%)	37 (84.1%)	12 (75.0%)
Education level				
Some high school but not completed	1 (0.7%)	0 (0%)	2 (4.6%)	0 (0%)
High school graduate or GED	24 (16.0%)	6 (7.9%)	4 (9.1%)	2 (12.5%)
Vocational, trade, or business school after high school	6 (4.0%)	4 (5.3%)	0 (0%)	1 (6.3%)
Some college, but did not receive a degree	30 (20.0%)	14 (18.4%)	7 (15.9%)	3 (18.8%)
Finished a 2-year college program	18 (12.0%)	12 (15.8%)	6 (13.6%)	1 (6.3%)
Finished a 4- or 5-year college program	51 (33.8%)	27 (35.5%)	18 (40.9%)	8 (50.0%)
Master's degree	17 (11.3%)	9 (11.8%)	5 (11.4%)	1 (6.3%)
PhD, MD, or other advanced degree	4 (2.7%)	4 (5.3%)	2 (4.6%)	0 (0%)
Marital status				
Currently married	79 (52.3%)	48 (63.2%)	29 (65.9%)	10 (62.5%)
Divorced	13 (8.6%)	10 (13.2%)	9 (20.5%)	6 (37.5%)
Living with someone as if you were married	14 (9.3%)	7 (9.21%)	1 (2.3%)	0 (0%)
Never married	36 (23.8%)	7 (9.21%)	5 (11.4%)	0 (0%)
Separated	4 (2.7%)	1 (1.32%)	0 (0%)	0 (0%)
Widowed	5 (3.3%)	3 (3.95%)	0 (0%)	0 (0%)
Smoking history				
Have never smoked	42 (27.8%)	21 (27.6%)	12 (27.3%)	8 (50.0%)
Currently smoking	7 (16.3%)	1 (4.6%)	2 (8.3%)	0 (0%)

^a^Values are *n* (%) unless otherwise stated. BMI, body mass index; GED, high school equivalency diploma.

**Table 2.** Operative Characteristics and Follow-up

Characteristic or follow-up	Augmentation		Reconstruction	
	Primary	Revisional	Primary	Revisional
	(*n* = 151)	(*n* = 76)	(*n* = 44)	(*n* = 16)
3-year follow-up	80.7%	78.9%	84.1%	87.5%
Placement, *n* (%)				
Subfascial	3 (2.0%)	0 (0%)	0 (0%)	0 (0%)
Subglandular	10 (6.6%)	9 (11.8%)	18 (40.9%)	2 (12.5%)
Submuscular	133 (88.1%)	67 (88.2%)	23 (52.3%)	9 (56.3%)
Other	5 (3.3%)	0 (0%)	3 (6.8%)	5 (31.3%)
Incision, *n* (%)				
Inframammary	117 (77.5%)	57 (75.0%)	22 (50.0%)	3 (18.8%)
Mastectomy scar	0 (0%)	0 (0%)	21 (47.7%)	10 (62.5%)
Periareolar	12 (7.9%)	7 (9.2%)	0 (0%)	1 (6.3%)
Mixed	1 (0.7%)	0 (0%)	1 (2.3%)	1 (6.3%)
Other	21 (13.9%)	12 (15.8%)	0 (0%)	1 (6.3%)
Implant texture, *n* (%)				
Smooth	144 (95.4%)	69 (90.8%)	44 (100%)	15 (93.8%)
Siltex	4 (2.6%)	6 (7.9%)	0 (0%)	0 (0%)
Missing	3 (2.0%)	1 (1.3%)	0 (0%)	1 (6.3%)
Implant volume (cc)				
Median	410	450	490	490
Standard deviation	100	148	138	101
Minimum/maximum	240/790	240/790	235/790	325/645

### Complications and Reoperations

Kaplan-Meier estimated cumulative incidence rates at 3 years for key complications in MemoryGel Xtra breast implant participants are provided in Table 3. Three-year follow-up data were available for 80.7% of the primary augmentation cohort, 78.9% of the revisional augmentation cohort, 84.1% of the primary reconstruction cohort, and 87.5% of the revisional reconstruction cohort.

**Table 3.** Three-Year Kaplan-Meier Estimated Cumulative Incidence Rates of Occurrence of Key Complications

Complications	Augmentation				Reconstruction			
	Primary		Revisional		Primary		Revisional	
	(*n* = 151)		(*n* = 76)		(*n* = 44)		(*n* = 16)	
	%	95% CI	%	95% CI	%	95% CI	%	95% CI
Key complications								
Any reoperation	9.7	(5.8, 16.2)	8.6	(3.9, 18.1)	19.2	(10.1, 34.8)	7.1	(1.0, 40.9)
Implant-related reoperation^a^	1.5	(0.4, 5.8)	2.8	(0.7, 10.7)	12.0	(5.2, 26.5)	0	NA
Capsular contracture Baker grade III/IV	1.5	(0.4, 5.8)	3.0	(0.8, 11.6)	7.3	(2.4, 21.0)	7.1	(1.0, 40.9)
Explantation with or without replacement	2.3	(0.7, 6.9)	4.3	(1.4, 12.9)	12.3	(5.3, 27.1)	0	NA
Explantation with replacement with study device	2.3	(0.7, 6.9)	2.9	(0.7, 11.1)	10.1	(3.9, 24.8)	0	NA
Rupture (suspected or confirmed)	0	NA	0	NA	2.5	(0.4, 16.5)	0	NA
Infection	0	NA	0	NA	0	NA	0	NA
Other complications^b^								
Asymmetry	2.3	(0.7, 6.9)	0	NA	0	NA	0	NA
Hypertrophic scarring	2.3	(0.7, 6.8)	0	NA	0	NA	0	NA
Delayed wound healing	0.8	(0.1, 5.3)	0	NA	2.4	(0.3, 16.1)	0	NA
Implant malposition/displacement	0	NA	0	NA	0	NA	0	NA
Erythema/inflammation	0.8	(0.1, 5.3)	0	NA	0	NA	0	NA
Implant size change	0.8	(0.1, 5.4)	0	NA	0	NA	0	NA
Nipple reduction	0.8	(0.1, 5.2)	0	NA	0	NA	0	NA
Suicide	0.8	(0.1, 5.2)	0	NA	0	NA	0	NA
New diagnosis of cancer, other than breast	0.7	(0.1, 4.6)	4.5	(1.5, 13.4)	0	NA	0	NA
Hematoma	0.7	(0.1, 4.6)	1.3	(0.2, 9.0)	2.4	(0.3, 15.7)	0	NA
Extrusion	0.7	(0.1, 5.1)	0	NA	0	NA	0	NA
Patient dissatisfaction with appearance	0	NA	1.5	(0.2, 10.0)	4.9	(1.2, 18.1)	0	NA
Nonhypertrophic scarring	0	NA	1.5	(0.2, 10.0)	0	NA	0	NA
Capsular contracture Baker grade II	0	NA	0	NA	4.9	(1.2, 18.1)	7.1	(1.0, 40.9)
Breast cancer recurrence	0	NA	0	NA	2.4	(0.3, 16.1)	0	NA
Wrinkling	0	NA	0	NA	2.4	(0.3, 16.1)	0	NA

^a^Implant-related reasons for reoperation include capsular contracture, rippling, infection, hematoma/seroma, and rupture. ^b^All other reported complications. CI, confidence interval; NA, not applicable.

#### Primary Augmentation

Any reoperation was estimated to have occurred for 9.7% of primary augmentation patients. The “any reoperation” data set included any secondary procedure in the vicinity of the breast, such as patient-requested reoperations including implant size change and patient dissatisfaction with appearance, as well as contralateral breast procedures and staged reconstruction procedures. The rate of implant-related reoperation in the primary augmentation cohort was 1.5% (where implant-related reoperations were defined as capsular contracture, wrinkling/rippling, infection, hematoma/seroma, and rupture—see discussion for further explanation). The most frequent complications in this cohort were explantation (2.3%, all of which were replaced with a study device), asymmetry (2.3%), hypertrophic scarring (2.3%), and Baker Grade III/IV capsular contracture (1.5%). Delayed wound healing, erythema/inflammation, implant size change, nipple reduction, and suicide each occurred at a rate of 0.8%. New diagnosis of non–breast cancer, hematoma, and extrusion each occurred at a rate of 0.7%. No other complications, including rupture, infection, implant malposition/displacement, and wrinkling/rippling, were reported through 3 years of follow-up.

#### Revisional Augmentation

The Kaplan-Meier estimate for any reoperation was 8.6%, with implant-related reoperations at 2.8%. The most frequent complications in this cohort were a new diagnosis of non–breast cancer (4.5%), explantation with or without replacement (4.3%, with 2.9% being replaced with a study device), Baker Grade III/IV capsular contracture (3.0%), patient dissatisfaction with appearance (1.5%), nonhypertrophic scarring (1.5%), and hematoma (1.3%). No other complications, including rupture, infection, implant malposition/displacement, and wrinkling/rippling, were reported through 3 years of follow-up.

#### Primary Reconstruction

The Kaplan-Meier estimate for any reoperation was 19.2%, with implant-related reoperations at 12.0%. The most frequent complications in this cohort were explantation with or without replacement (12.3%, with 10.1% being replaced with a study device), Baker Grade III/IV capsular contracture (7.3%), patient dissatisfaction with appearance (4.9%), rupture (2.5%), wrinkling/rippling (2.4%), recurrence of breast cancer (2.4%), delayed wound healing (2.4%), and hematoma (2.4%). No other complications were reported through 3 years of follow-up.

#### Revisional Reconstruction

The Kaplan-Meier estimate for any reoperation was 7.1%, with no implant-related reoperations. The rate of Baker Grade III/IV capsular contracture was 7.1%, as was the rate of Baker Grade II capsular contracture. No other complications were reported through 3 years of follow-up.

### Effectiveness

**Figure 1.**
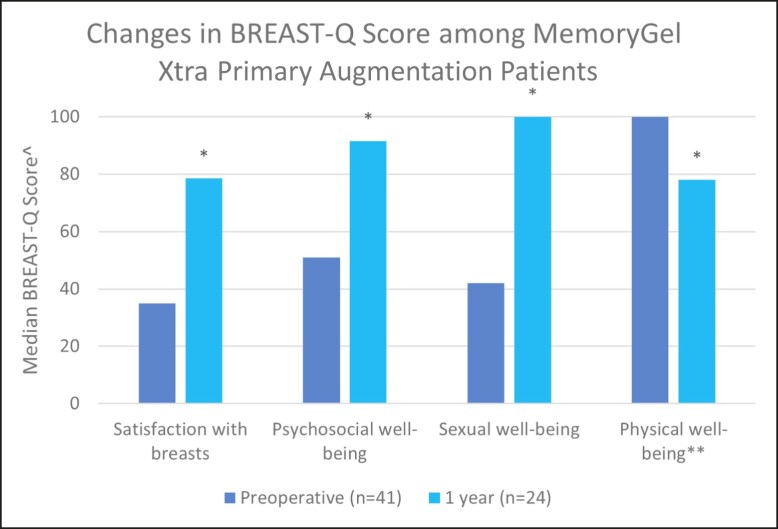
Changes in BREAST-Q score among MemoryGel Xtra primary augmentation patients. ^ Higher values represent more favorable outcome; * = *P* < .0001, based on the participants who have both preoperative and postoperative data; ** the large majority of cases (88%) were submuscular augmentations, which, as noted by Alderman et al “are associated with a delay in recovery of physical functioning.”^2^

**Figure 2.**
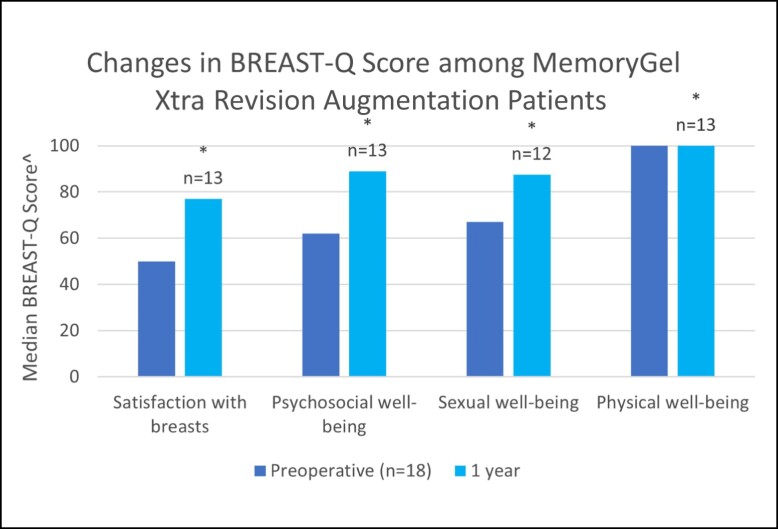
Changes in BREAST-Q score among MemoryGel Xtra revisional augmentation patients. ^ Higher values represent more favorable outcome; * = *P* < .0001.

**Figure 3.**
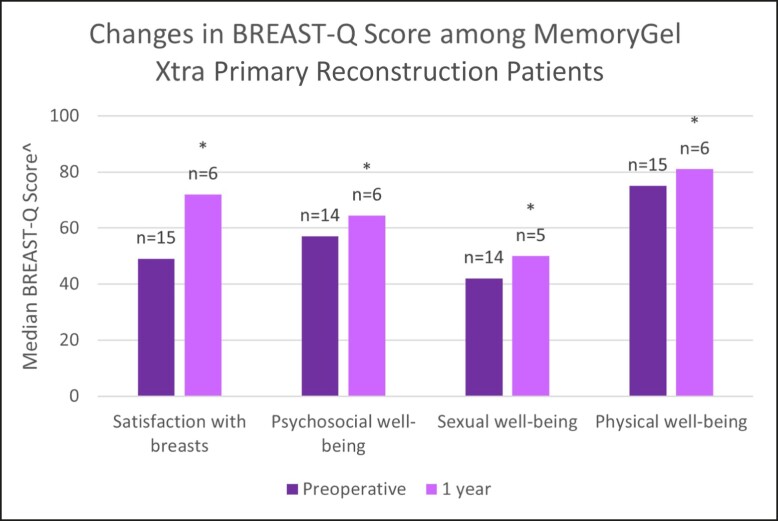
Changes in BREAST-Q score among MemoryGel Xtra primary reconstruction patients. ^ Higher values represent more favorable outcome; * = *P* < .0001.

**Figure 4.**
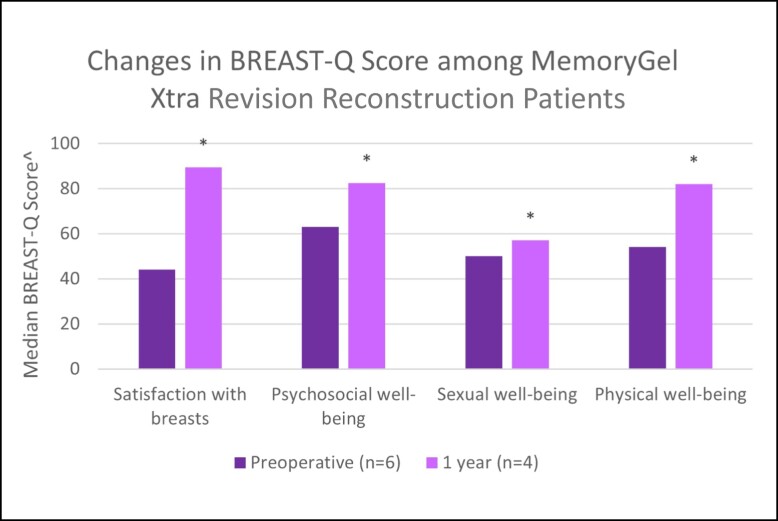
Changes in BREAST-Q score among MemoryGel Xtra revisional reconstruction patients. ^ Higher values represent more favorable outcome; * = *P* < .0001.

Because BREAST Q data is being collected preoperatively and at, 1, 5, and 10 years postoperatively, data for the preoperative and 1-year postoperative questionnaires were reported here. Preliminary measured BREAST-Q score values before operation (baseline) and at the 1-year follow-up point are presented in Figure 1 and Table 4. In MemoryGel Xtra primary augmentation patients, satisfaction with breasts, psychosocial well-being, and sexual well-being all increased significantly from baseline (*P* < .001), whereas physical well-being was significantly lower. All median BREAST-Q score changes improved in the other cohorts aside from physical well-being in the revisional augmentation cohort, which scored maximally both preoperatively and at 1 year postoperatively (Figures 2-4, Table 4).

**Table 4.** BREAST-Q Scores for MemoryGel Xtra Patients

Cohort	Time point	Statistic	Satisfaction with breasts	Psychosocial well-being	Sexual well-being	Physical well-being
Primary augmentation	baseline	*n*	41	41	41	41
		median	35	51	42	100
	1 year	*n*	24	24	24	24
		median	78.5	91.5	100	78
Revisional augmentation	baseline	*n*	18	18	18	18
		median	50	62	67.0	100
	1 year	*n*	13	13	12	13
		median	77	89	87.5	100
Primary reconstruction	baseline	*n*	15	14	14	15
		median	49	57	42	75
	1 year	*n*	6	6	5	6
		median	72	64.5	50	81
Revisional reconstruction	baseline	*n*	6	6	6	6
		median	44	63	50	54
	1 year	*n*	4	4	4	4
		median	89.5	82.5	57	82

**Figure 5.**
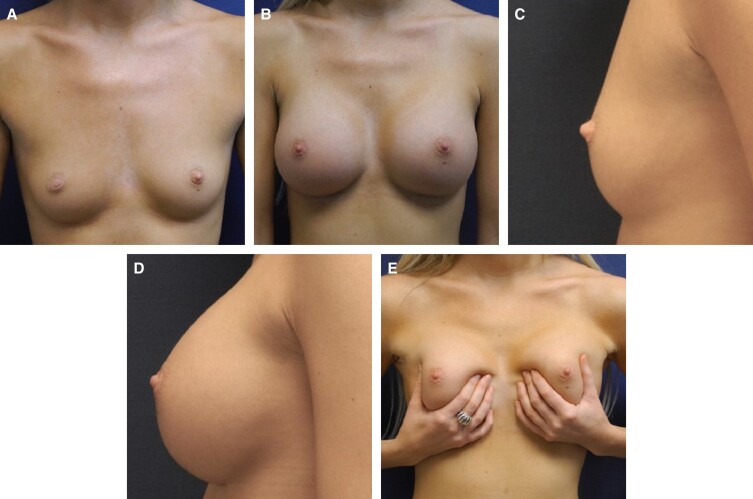
Primary breast augmentation in a 23-year-old female with MemoryGel Xtra Moderate Plus Profile implants (325 cc). (A, C) Photographs taken preoperatively; (B, D) photographs taken 2 years postoperatively; and (E) photograph taken 2 years postoperatively displaying Baker grade I capsules.

**Figure 6.**
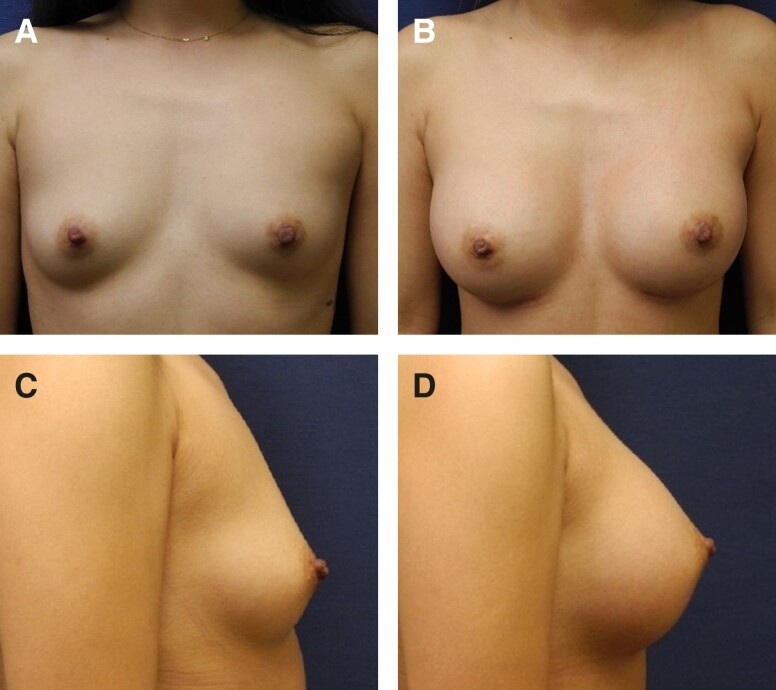
Primary breast augmentation in a 27-year-old female with MemoryGel Xtra Moderate Plus Profile implants (325 cc). (A, C) Photographs taken preoperatively; and (B, D) photographs taken 2 years postoperatively.

**Figure 7.**
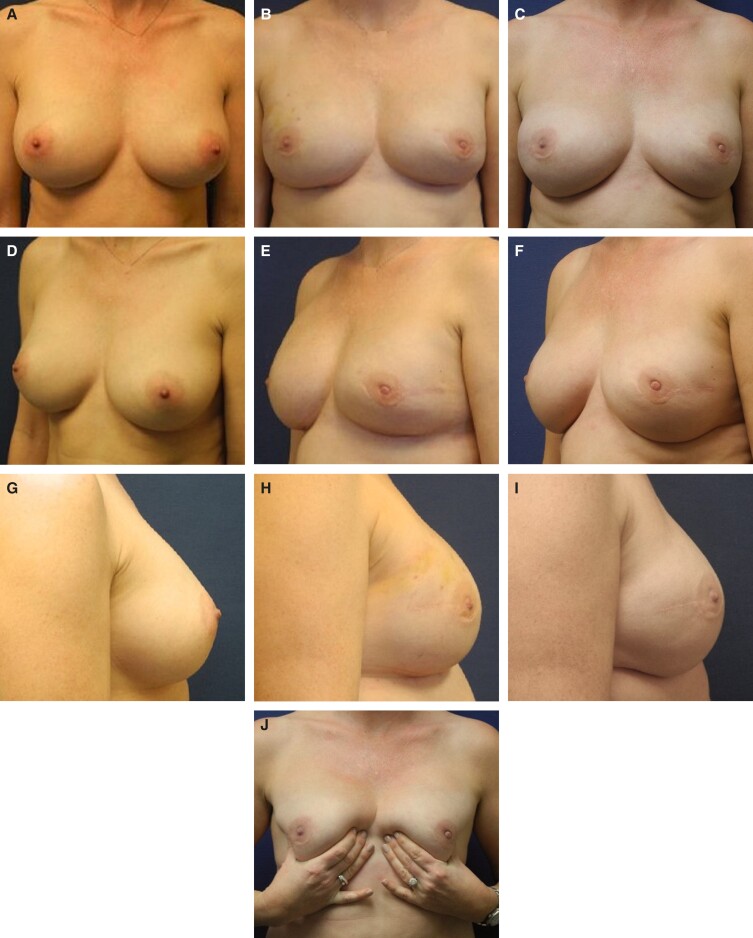
Primary breast reconstruction with MemoryGel Xtra. Planned bilateral mastectomy with 2-stage reconstruction in a 43-year-old female utilizing MemoryGel Xtra Moderate Plus Profile implants (465 cc). (A, D, G) Photographs taken preoperatively; (B, E, H) photographs taken 5 weeks postoperatively; (C, F, I) photographs taken 8 months postoperatively; and (J) photograph taken 1 year postoperatively displaying Baker grade I capsules.

**Figure 8.**
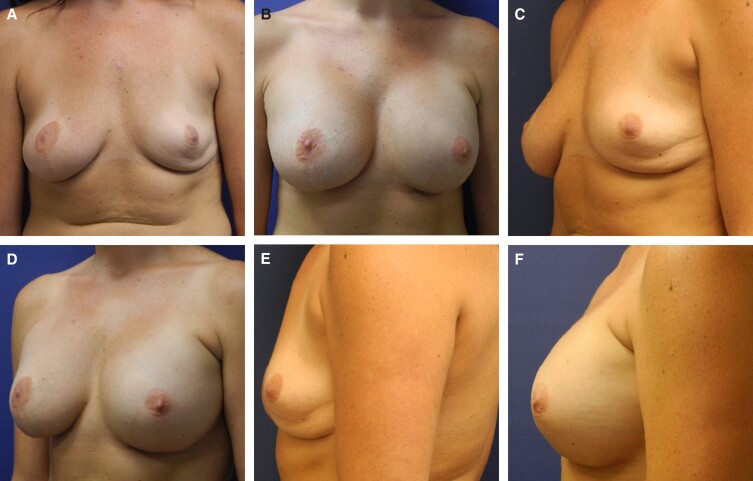
Revisional breast augmentation in a 27-year-old female with MemoryGel Xtra Moderate Plus Profile implants (405 cc). Patient presented with infected left breast and right implant ruptured. Both implants were removed and replaced. (A, C, E) Photographs taken preoperatively; and (B, D, F) photographs taken 1 year postoperatively.

**Figure 9.**
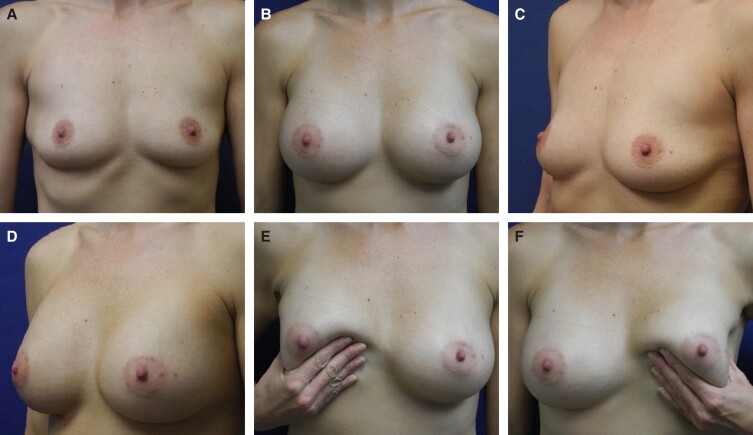
Primary breast augmentation in a 40-year-old female utilizing MemoryGel Xtra Moderate Plus Profile implants (370 cc). (A, C) Photographs taken preoperatively; (B, D) photographs taken 1 year postoperatively; and (E, F) photographs taken 1 year postoperatively displaying Baker grade I capsules.

Representative photographs of females before surgery and 1 to 2 years postimplantation with MemoryGel Xtra devices are shown in Figures 5-9. Figures 6 and 7 display actual study patients whereas the other photographs are of non–study patients.

### BIA-ALCL and SCC

No reports of BIA-ALCL or SCC have been reported for patients implanted with MemoryGel Xtra devices in the study. During the conduct of the study, there have been no reports of BIA-ALCL or SCC. However, 1 case of BIA-ALCL was reported in the medical history of a patient undergoing revisional reconstruction with textured, MemoryShape implants before enrollment in the study. The patient completed treatment and and was disease-free prior to entry into the study. As of the most recent follow-up with the patient in 2022, there is no recurrence of BIA-ALCL.

### Death

In the primary augmentation MemoryGel Xtra cohort, 1 patient has been reported as deceased, by suicide. In the rest of the study, 1 death in a patient implanted with MemoryGel breast implants was also reported. Despite repeated follow-up, no further information is available, and the cause of death is unknown. No other deaths have been reported in the study.

## DISCUSSION

This analysis of the MemoryGel Xtra cohort from the MemoryGel and MemoryShape Combined Cohort Clinical Study is the first published clinical data from a prospective study on this novel innovative addition to the Mentor MemoryGel implant portfolio. MemoryGel Xtra breast implants are designed to reduce the incidence of implant-related wrinkling/rippling and its influence on capsular contracture and shell failure through optimal shell-gel filling. They are yet another differentiated implant option in the plastic surgeon's arsenal, providing improved central implant projection when such an influence on shape is desired. In the authors’ experience, these design elements make MemoryGel Xtra of particular use in females with thin skin envelopes and/or limited soft tissue coverage but are similarly ideal for a wide variety of patients.

MemoryGel Xtra devices show low complication rates based on 3-year follow-up on over 81% of 287 females who were implanted with the devices, including 151 primary augmentation participants. Cumulative estimated risks through 3 years are presented in Table 3. Risks greater than 1% in the primary augmentation cohort were as follows: 9.7% for any reoperation, 2.3% explantation (all of which were replaced with study devices), 2.3% asymmetry, 2.3% hypertrophic scarring, and 1.5% capsular contracture (Baker Grade III/IV). Of note, there were no reports of malposition/displacement, infection, rupture, or wrinkling/rippling.

While the study to date has reported complication rates by device type too low to allow meaningful comparisons between device types with different silicone gel fill levels, experience suggests that by increasing the degree to which implant shells are filled, one may reduce the incidence of clinically significant wrinkling/rippling.^3–6^ Furthermore, experience with saline-filled implants suggests that by maximally filling (or over-filling) these devices, there was a reduced incidence of wrinkling/rippling as well as a prolongation of implant lifespan thought to be due to a reduced incidence of shell redundancy–related fold-flaw failure. This concept was incorporated into the design for MemoryGel Xtra implants: gel fill was optimized with the intention of increasing central device fullness and projection as well as reducing surface wrinkling/rippling, all of which could potentially lead to an enhanced implant lifespan. Continued close observation of clinical and performance outcomes in the study over the next 7 years is expected to demonstrate whether the precision-filled MemoryGel Xtra devices produce similar long-term results. Breast implant reoperations are sometimes requested by patients or suggested by surgeons based on a desire to improve aesthetic outcomes rather than to address medical complications. As others have suggested and we explained previously, implant-related complications (capsular contracture, wrinkling/rippling, infection, hematoma/seroma, and rupture) resulting in reoperation may be more informative to providing an understanding of implant safety and effectiveness than merely reporting all reoperations.^7^ Although the cumulative estimated risk of secondary operations for any cause in the breast vicinity in the primary augmentation cohort was 9.7% at 3 years, the risk for implant-related reoperation in this same time frame was only 1.5% (Table 3).

The clinical effectiveness of MemoryGel Xtra was demonstrated via BREAST-Q patient-reported outcome measures. Primary augmentation participants reported significantly higher satisfaction with breasts, psychosocial well-being, and sexual well-being 1 year after implantation. Although a reduction in physical well-being was noted in this cohort, this is possibly attributable to a lingering postoperative effect, because 88% of these cases were submuscular augmentations which are known to be “associated with a delay in recovery of physical functioning.”^2^ The study in which this was concluded looked at BREAST-Q scores out to 6 months and concluded that physical functioning does appear to be negatively affected by submuscular breast augmentation, although the negative effect is diminished at 6 months compared with 6 weeks. Unfortunately, in the study data were not collected beyond 6 months and therefore were unable to determine when or if physical well-being scores of females undergoing submuscular breast augmentation recovered to baseline levels. The current study follows different patients in different numbers at a different time, with mostly different plastic surgeons, so directly comparing values and recovery time between the 2 studies is probably not valid. What we can say is that patient-reported physical well-being of females undergoing (predominantly) submuscular breast augmentation in both studies have yet to recover to baseline levels by the latest date measured thus far. Along with the other BREAST-Q areas, we are continuing to monitor physical well-being to evaluate when/if physical function returns to baseline, because the 1-year results are most likely a function of the implant pocket, not the implant itself. The 5- and 10-year patient-reported outcome results will be reported in a subsequent publication. The medians of each of these well-being measures improved from preoperative to 1 year postoperatively in the other cohorts, aside from physical well-being in the revisional augmentation cohort, in which it scored maximally at each time point.

Limitations of this analysis include the nature of follow-up data collection. Although follow-up visits are to be scheduled per the site's standard of care, the only requirement for annual follow-up is that the study participants fill out online questionnaires. It is therefore possible that complications have occurred that were not reported or, inversely, that some complications were reported inaccurately. In the case of reoperations, however, the surgeon is involved in reporting. In addition, lengthier follow-up is needed to accurately assess long-term complications, such as rupture.

## CONCLUSIONS

In conclusion, this report on 3 years of follow-up data, the first published prospective clinical data on Mentor MemoryGel Xtra breast implants, supports the safety and effectiveness of MemoryGel Xtra breast implants. Comparisons of outcomes by various implants used in this study will be the subject of subsequent analyses, to examine benefits of MemoryGel Xtra devices as well as provide additional comparative data on other Mentor breast implants included in the study.
